# The economic impact of non-communicable diseases among households in South Asia and their coping strategy: A systematic review

**DOI:** 10.1371/journal.pone.0205745

**Published:** 2018-11-21

**Authors:** Anupa Rijal, Tara Ballav Adhikari, Jahangir A. M. Khan, Gabriele Berg-Beckhoff

**Affiliations:** 1 Young Earth, Kathmandu, Nepal; 2 Nepal Development Society, Chitwan, Nepal; 3 Department of Public Health, Aarhus University, Aarhus, Denmark; 4 Liverpool School of Tropical Medicine, Liverpool, United Kingdom; 5 Department of Learning, Informatics, Management and Ethics, Karolinska Institutet, Sweden; 6 Unit for Health Promotion Research, University of Southern Denmark, Denmark; RTI International, UNITED STATES

## Abstract

**Background:**

Out of pocket payment (OOPP), is the major health financing mechanism in South Asia region. With the rising burden of non-communicable diseases (NCDs), the region is facing a high financial burden. However, the extent and nature of economic impact caused by treatment and management of NCDs at the household level is yet unknown.

**Method:**

We conducted a systematic review using Medline and Embase databases. Only peer-reviewed quantitative studies published between January 2000 to December 2016 assessing OOPP or catastrophic health expenditure or impoverishment or financial coping strategy due to at least one of the four major NCDs—cardiovascular diseases(CVDs), diabetes, cancer, chronic respiratory disease in South Asia region was included in the review. The review is registered in PROSPERO no: CRD42017059345.

**Results:**

A total of 21 studies (of 2693 records identified) met the inclusion criteria. The economic impact was most frequently studied in CVDs and in terms of OOPP. The studies collectively indicated high OOPP, higher likelihood of catastrophic expenditure and impoverishment for inpatient care for these major NCDs which was visible in all income levels. Borrowing and selling off assets were the most common forms of coping strategies adopted and varied inconsistently between urban and rural households. The true extent of the economic impact, however, remains difficult to determine due to methodological heterogeneity regarding outcomes reported and measures employed for calculation of OOPP, catastrophic expenditure, and impoverishment across these four major NCDs and between nations.

**Conclusion:**

The economic impact due to treatment and management of CVDs, diabetes, cancer and chronic respiratory diseases among households in South Asia seems dire. Given the lack of sufficient evidence the review stresses the need for further research in the region to develop evidence-informed nationally tailored prepayment mechanisms covering NCDs to reduce economic vulnerability and standardization of tools measuring the economic impact for generating comparable estimates.

## Introduction

Globally, cardiovascular diseases (CVDs), cancers, chronic respiratory diseases (chronic obstructive pulmonary disease and asthma) and diabetes are leading non-communicable diseases (NCDs) contributing 81% of all NCDs related mortality [1]. NCDs also accounts for 58% of the Disability Adjusted Life Years (DALYs) [1]. With rapid urbanization, sedentary lifestyle [2], increased consumption of unhealthy diets, high alcohol use, and high blood pressure the burden of NCDs have escalated throughout the world in between 1990 and 2010 so as in South Asia [3, 4]. The South Asian countries namely Afghanistan, Bangladesh, Bhutan, Maldives, Nepal, India, Pakistan and Sri Lanka, which are mostly low and middle-income countries with regional Gross Domestic Product (GDP) per capita 1639.7 United States Dollar (USD) and home to a quarter of world population [5]. The region currently bears a high burden of NCDs, and related death is expected to increase by 20% in the World Health Organization (WHO)- South-East Asia Region [6]. Studies suggest that the manifestation of CVDs and onset of diabetes is much earlier among South Asians than other ethnicities due to adverse metabolic factors requiring longer-term medication [7].

The growing incidence of NCDs threatens the already weakened health system in South Asia [7, 8]. In the absence of adequate policy direction, diagnostic capacity and effective organizational measures; addressing these emerging NCDs will have a far-reaching impact on health care, both at the individual and institutional level in South Asia. People will be required to pay for expensive treatments and medications out of pocket as most of the countries in the region do not have a functional population-wide insurance system. Financing for health depends heavily on out of pocket payment (OOPP) [9] in the region, which is a derogatory form of health financing. OOPP increases households cost associated with healthcare and forces households to unprecedented financial catastrophe and impoverishment [10]. WHO defines catastrophic health expenditure as the health expenditure greater than or equal to 40% of a household's effective income remaining after basic subsistence needs have been met (capacity to pay). If catastrophic expenditure pushes a household below this income threshold the poverty line it is known as impoverishment [11]. In this article, the term economic impact collectively refers to the impact caused by OOPP, catastrophic health expenditure, impoverishment or any other indirect costs and financial burden incurred due to management and treatment of CVDs, diabetes, chronic respiratory diseases and cancers. Individuals are not very price sensitive when it comes to treatment and management of disease like NCDs which triggers premature death or disabilities if not treated timely [12]. Hence, households despite their incapability to pay for the health-care services undergo catastrophic payment or adopt different coping behaviors to meet the financial need for hospitalization and health care costs [13]. Whereas, this economic constraint may also lead to the number of untreated cases eventually increasing the burden of NCDs.

The quarter of world population (where 15.1% of the population live under 1.90 USD per day) [5] living in this region are on the verge of slipping into a vicious cycle of poverty and impoverishment while seeking for healthcare services specially for NCDs. This compromises the attainment of the global goal of Universal Health Coverage which is target 8 of goal number 3 of Sustainable Development Goals (SDG) [14]. This goal calls for grand convergence to provide health services relative to need and ensuring financial protection so that health care is within reach of all the population. It also further challenges the attainment of the global action plan for prevention and control of NCDs 2013–2020 which deals with relative reduction of premature NCDs deaths by 25% by 2025 and reducing the contribution of NCDs in financial impoverishment, by identifying the NCDs which needs the urgent intervention at the country level [15].

The current literatures on the discourse of assessing financial burden of NCDs in the low and middle-income countries by Kankeu et al. [16] and Gupta et al.[17] emphasize the need for prioritizing robust research on estimating costs incurred and impoverishment effect due to NCDs. It is important to carry out new research to produce evident knowledge in the resource constraint settings. The need for further research in low and middle-income countries was also concluded by Jaspers et al. in a systematic review assessing global impact of NCDs and impoverishment which included eight studies assessing the economic impact of NCDs from India and two from Pakistan both being South Asian countries [18]. A review of literature by Saksena et al. discussed the impact of out of pocket payments for non-communicable diseases in developing countries which also included some studies from India suggested that household share a substantial proportion of income for NCDs treatment specially hospitalization related expenditures [19]. However, a comprehensive assessment of the economic impact caused by specific NCDs exclusively in South Asia region is still missing so far. Hence, given the gap in the literature, and to update on existing evidence of economic impact by NCDs the current study will systematically review the existing evidence on the OOPP, catastrophic and impoverishment effect of NCDs along with individual and households coping strategies to these financial constraints in South Asia.

## Methods

In order to conduct a systematic review assessing the economic impact of NCDs and impoverishment among households in South Asia region, the Preferred Reporting Items for Systematic Reviews and Meta-analyses statement was used as a reporting guideline for this review. The PROSPERO registration number for the review is CRD42017059345.

### Search strategy

We systematically searched two electronic databases accessed through Ovid: Medline and EMBASE by using database tailored search strategy. The search strategy was adapted from the similar systematic review conducted by Jaspers et. al.[18] and was based on PECO (Population, Exposure Comparison, and Outcome) framework covering the objectives of this review and appropriate Subject Headings was used and was searched in titles, abstracts, topics, and keywords depending on the database. The latest search was conducted on 17^th^ February 2017. Additionally, snowballing technique was applied for the manual search of studies from the list of references and citations of retrieved articles to identify studies not found in the database search. The complete search strategy is available as supporting document **(S1 Table).**

### Inclusion criteria

Quantitative studies conducted in at least one of the following countries: Nepal, India, Pakistan, Sri Lanka, Bhutan, Bangladesh, Maldives among any gender (male or female) in any age group including at least one of the four major NCDs: CVDs, type II diabetes, chronic respiratory diseases (chronic obstructive pulmonary disease and asthma) and cancers examining at least one of the measures of economic consequences caused by NCDs at households were considered in the review. In this systematic review, we only included peer-reviewed English research articles published between January 2000 to December 2016.

Given the existence of different types of cancers and our limited resources, the detailed search strategy in the review majorly focused on cancers with leading DALYs rate among South Asian male (Lung cancer and Oral Cancer) and female (Breast cancer and Cervical cancer) [20]. However, studies assessing the economic impact of these specific cancer or cancers/neoplasm in general were both included in the review. The economic measures included in the review were direct costs, indirect costs, expenditure on medicine, transport, out of pocket expenditure, financial hardship, catastrophic health expenditure, impoverishment, individual or household cost, poverty line, or coping strategy for NCDs related financial burden.

### Exclusion criteria

Studies though satisfying the inclusion criteria but with an inadequate assessment of measures of outcome, and or of unsatisfactory quality and unfeasible for data extraction were not considered for the review. Studies from Afghanistan was not considered for the review as it became the member of South Asia only since 2008.

### Study selection

Studies were initially identified based on title and abstracts, and when abstracts were not relevant or did not provide sufficient information, the full-text articles were retrieved and screened against inclusion and exclusion criteria by first and second author independently. Any disagreement between two reviewers was resolved through consensus and consultation of the third reviewer.

### Data extraction from selected articles

All the references form both the databases were exported to EndNote X7.7.1, and duplicate studies were removed. Three different data collection forms were made to collect relevant information from the included articles. The first form included information about the characteristics of studies. The second data extraction form was used for category wise assessing quality of the study based on the Newcastle–Ottawa Quality Assessment Scale and final quality score for each study was assigned [18]. The third data extraction form contained the details about the assessment of the outcome of interest.

Local currencies were converted to US dollars (USD) to enhance comparability between the eligible studies. We used country specific Purchasing Power Parity conversion rate provided by World Bank data [21]. The conversion rate of the publication year of the study was used. Furthermore, all USD were converted to dollars of 2016 using the consumer price index conversion factors [22].

### Risk of bias analysis

Newcastle–Ottawa Quality Assessment Scale (NOS) adapted for the cross-sectional and descriptive study was used in the review [23]. The NOS scale assesses the quality of the articles in three domains of selection, comparability, and exposure and is based on ‘star system.’ The selection and exposure category include four and three items respectively and can be provided one star each while comparability with one item can be provided two stars. Hence the NOS scale can have maximum nine stars for the highest quality. A score was tallied by adding up the stars. A study was categorized as being of low risk of bias or highest quality if a total of 8 to 9 stars were allocated, medium risk of bias if 6 to 7 stars were allocated and of high risk or poor quality if the total score awarded was ≤ 5 stars.

### Data analysis and synthesis

Disease-specific data extracted were synthesized in groups and inferences were made. Given the heterogeneity regarding methods and outcomes addressed, the results were not combined across studies, and no summary measures were calculated.

## Result

**Fig 1:**
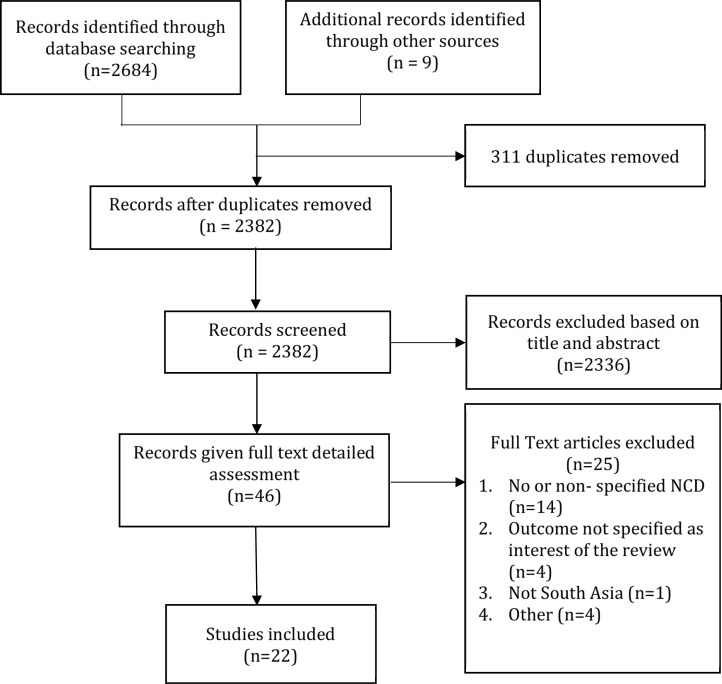
PRISMA flow-chart for systematic review of studies.

From 2,693 references initially screened 22 studies met the inclusion criteria and was included in the review as shown in **Fig 1**

### Characteristics of the study reviewed

**Table 1** shows characteristics of 21 studies included in the review out of which 14 studies were solely based in India. There were no studies identified from Maldives and Bhutan regarding economic impact, related impoverishment and coping strategy due to NCDs. Studies varied from 50 to 200,000 observations. Fifteen studies included individuals as sample unit while eight studies used household as a unit of analysis and all these studies had sample size >1500 [24–31] [24, 25, 29–34].

**Table 1:** Characteristics of the studies.

Study Design	Location	Period of Surveillance	Sampling	Number in analysis	Gender	Age	Reported NCD	Source, Publication year
**Cohort**	India	Apr 2009-Oct 2011	Purposive	189 ind	Both	18 yrs and above	Stroke	Kwatra et al, 2013 [36]
		Mar 2013-Jul 2014	Purposive	644 ind	Both	0 to 18	Congenital Heart Disease	Raj et al, 2015 [37]
		Jun 2011-May2012	Purposive	1635 ind	Both	18 yrs and above	Acute Coronary events	Jan et al, 2016 [35]
**Cross- sectional**	India	1995–96 and 2004	Random	200000 hld	NA	NA	Diabetes, Heart Disease, Cancer, Bronchial Asthma	Engelgau et al., 2012[25]
		2004	Random	74 000 hld	NA	All ages	Cardiovascular disease	Karan et al, 2014 [28]
		Jun-Sep 2008	Random	210 ind	Both	25–70 yrs	Acute Coronary syndrome	Davidanam et al, 2012[42]
		2008–2009	Random	500 ind	Both	25–70 yrs	Cardiovascular disease	Huffman et al, 2011[44]
		Jan and Jun2004	Random	Diabetes: 438, CVD: 2129 ind	Both	NA	Diabetes, Cardiovascular disease	Roa et al, 2011 [38]
		NR	Purposive	50 ind	Both	20–50 yrs	Diabetes	Grover et al, 2005 [46]
		NR	Purposive	596 ind	Both	NA	Diabetes	Shobhana et al, 2000 [45]
		2004	Random	73000 hld	Both	NA	Cancer, Cardiovascular disease, Diabetes	Joe et al, 2015 [33]
		2004	Random	74000 hld	NA	All ages	Cancer	Mahal et al,2013 [29]
		NA	Random	199 ind	Both	NA	Stroke	Das et al, 2010 [39]
		Mar–May 2011	Random	508 ind	Both	NA	Cancer	Nair et al, 2013 [48]
	Bangladesh	2012–2013	Random	476 ind	Both	≥ 20 yrs	COPD	Uddin et.al,2014[60]
		Feb-Apr 2010	Purposive	166 ind	Both	18+ yrs	Diabetes	Joshi et. al, 2012[40]
		2009	Purposive	3941 hld	NA	NA	Diabetes, Heart Disease, Cancer, Asthma	Hamid et al, 2014 [26]
		Aug-Nov 2011	Random	1593 hld	NA	NA	Heart Disease, Asthma	Rahman et al, 2013 [30]
	Pakistan	2009–2010	Purposive	67 ind	Female	NA	Breast Cancer	Zaidi et al, 2012 [41]
		Jul to Sep 2006	Random	345 ind	Both	20–60 yrs	Diabetes	Khowaja et al, 2007 [47]
	Nepal	Nov 2011- Jan 2012	Random	1997 hld	NA	NA	Diabetes, Asthma, Heart Disease	Saito et. al, 2014[31]
	Nepal, Srilanka, Bangladesh, India	2002–2003	Random	Ban:5942, Ind:10692, Nep:882, SriL:6805 hld	NA	18+ yrs	Angina	Alam et al, 2014 [24]

Ind: individuals, hld: households, yrs: Years, NA: Not Available

Majority of the studies were cross-sectional in nature. Only three studies followed up to six months after discharge of patients and collected information on economic impact [35–37].

Fifteen studies used random sampling while 9 studies were based on purposive sampling for identifying the respondents. The purposive sampling was usually done in purposively identified hospitals or health care center setting among patients visiting health centers or parents/closet patient party of either hospitalized patients or those who survived hospitalization/surgery except for one where purposive program area was first selected and household survey was done.

For assessment of NCDs, majority of the studies identified CVDs, diabetes, chronic respiratory diseases and cancers based on clinically confirmed/diagnosed record, inpatient/outpatient cases, hospitalization record, or those who survived hospitalization/surgery. Five studies in the review identified NCDs based on self-reporting of symptoms or ailments and cross-matching with the ICD-9 [25] or with categorization of disease based on symptoms reported by previous studies or WHO classification [26, 30, 38]. While one study self-reported symptoms were first cross-matched with pre-determined stroke definition and these positive cases were then confirmed neurologist [39]. Likewise, two studies used both clinically confirmed cases wherever information on clinical diagnosis was available if not self-reported symptoms cross matched with NCD pre-determined categorization was used [24, 31] while one study was entirely based on self-reporting of the conditions from respondents [40].

In all the studies included in the review, the measure of outcome, i.e., economic impact of NCD was based on self-reported information on household expenditure or cost or financial burden at the household or individual level. Two studies applied Propensity Score matching to assess the economic burden of NCD by comparing the means between the NCD affected households and matched control household using a t-test [24, 34]. Except one, all other NCDs were studied the among adult population 18 years and above. CVD was the most frequently reported NCDs in the studies. Overall, among the 21 studies included in the review, CVD was most commonly studied NCD (14 studies) followed by diabetes (9 studies), cancer (6 studies) and lastly chronic respiratory disease (5 studies). Except one study mentioning breast cancer, no other cancer articles included in the review have specified on the type of cancer assessed in the study and its economic impact on household [41].

Majority of the studies (12 studies) were of poor quality with scoring ≤ 5 stars, 7 were of moderate quality and only 3 studies were of high quality [24, 28, 29]. The median quality score was 5 out of 9 (minimum 1, maximum 8) with an interquartile range of 3. **(S2 Table)**

### Measurement of economic impact

The measurement of economic impact caused by NCDs was heterogeneous. The most common reported economic impact was OOPP followed by catastrophic health expenditure while very few studies dealt with impoverishment, financial hardship, and coping strategy.

### OOPP and financial burden

The common measure of OOPP was expenditure for per hospital stay or inpatient care or hospitalization cost per household member [24, 25, 34, 35, 38] while cost subdivided in terms of indirect cost, direct cost, non-medical cost or was calculated altogether [42, 43]. OOPP as the proportion of total household spending [34, 38] or perceived financial hardship by caretakers was also studied [41].

### Catastrophic health expenditure

Catastrophic health expenditure has been reported to varying degree of threshold and denominators. Among the studies included in the review, two studies included household’s ‘capacity to pay’ as denominator at 40% level threshold [24, 42] while Huffmann et al. used household non-food expenditure [44]. Likewise, studies also included annual baseline income and total household expenditure as denominator at 30% and 10% threshold respectively [31, 35]. Only one study measured the intensity of catastrophic payment by assessing the mean positive overshoot which uses only those households that have experienced catastrophic health expenditure in actual as the denominator [31].

### Impoverishment

Out of 22 studies, only three studies dealt with impoverishment effect of out of pocket spending on health care for the NCDs of interest [24–26]. Impoverishment was expressed as percentage of household healthcare spending exceeding purchasing power parity represented in terms of either relative poverty line USD 0.88 (for Bangladesh) [26] and absolute poverty line USD 1.25 per day per person [24]. Hamid et al. further represented medical impoverishment in terms of poverty impact, poverty gap and normalized poverty gap [26]. While Engelgeu et al expressed it in terms of odds of undergoing impoverishment for household with CVDs and cancer as compared to household with communicable disease and used relative poverty line estimates for different states and regions of India [25].

### Coping strategy

Only13 studies dealt with the coping strategy adopted by individuals and households to meet OOPP for NCDs related treatment. Studies reported either the percentage or risk of using alternate financial measures like borrowing money, selling of assets, taking loans also denoted as distress financing [30, 33].

### Summary of the economic impact of NCDs in South Asia

Overall, this systematic review highlighted that major non-communicable diseases, like cardiovascular disease, cancer, diabetes and chronic respiratory diseases incurred economic impact among households in South Asia, however, the extent and magnitude of the impact is still inconclusive. Nonetheless, the studies in the review uniformly showed OOPP, catastrophic health expenditure and impoverishment was higher among households with NCDs compared to household without NCDs. Furthermore, the economic impact was visible at different income levels. Borrowing and selling off assets were most commonly exhibited coping strategy by South Asian household. These coping behaviors however differed inconsistently based on place of residence either rural or urban.

### Economic impact of cardiovascular diseases

In **Table 2**, studies showed that out of pocket health spending per person was high among angina and CVDs affected household as compared to control and matched household respectively [24, 28]. The regional proportion of households suffering from financial catastrophe for CVD-related treatment ranged from 20% to 90% depending on the chosen income threshold taken in the studies. The inter-country study among Nepal, Bangladesh, India, and Sri Lanka revealed that household in Bangladesh had the highest prevalence of catastrophic health expenditure (as household’s capacity to pay at 40% cut off point) and impoverishment due to Angina treatment (39.4% and 12.6% respectively). Among Nepalese household, a significant proportion had to undergo borrowing or selling off assets (57.62%) to finance health expenditure. However, much higher (84%) catastrophic spending was reported for Acute Coronary Syndrome (ACS) treatment in India at the same threshold [42]. Studies reflected that the financial hardship and catastrophic health expenditure led by CVDs treatment was visible in both rich and poor households. More than a quarter (26.3%) of high-income Indian households had decreased income due to treatment for CVDs [44]. Likewise, the wealthiest household with heart disease from Nepal were 2.36 times more likely to undergo catastrophic health expenditure as compared to the household without heart disease [31]. Moreover, borrowing, contribution from friends, sell off assets to meet OOPP for CVDs treatment was found to be concentrated in rural areas in India. Raj et. al showed that such coping strategies were prominent even after completion of surgery for congenital heart surgery as more than half (52.1%) of households in India reported borrowing money even after 6 months of discharge from congenital heart surgery to take care of the child and pay the loan made earlier for the treatment [37]. **(Table 2)**

**Table 2:** Economic impact of cardiovascular disease among households in South Asia.

Study Design	Location	Type of Outcome	Outcome Specified as	Assessment Type	Point Estimate	Author
**Cohort**	India	OOPP	Per patient cost of hospitalizations due to ST elevated MI	Mean, $	2500	Jan[35]
		Catastrophic Expenditure	OOPP at 6 week> 30% of annual baseline household income, among insured patient	Percentage	20	
			OOPP at 6 week> 30% of annual baseline household income, among uninsured patient	Percentage	60	
		OOPP	Total hospital cost (Direct medical+ direct non-medical + indirect costs), at 6 months	Mean, $ (95% CI)	4962.31 (4467.51, 5452.44)	Kwatra[36]
			Direct medical cost, at 6 months	Mean, $ (95% CI)	3235.18 (2912.86, 3589.57)	
			Non-medical cost, at 6 months	Mean, $ (95% CI)	297.08 (253.74, 342.94)	
			Indirect cost, at 6 months	Mean, $ (95% CI)	1429.99 (1231.59, 1648.21)	
		OOPP	Total hospitalization cost (Direct+ Indirect costs), for surgery	Mean, $ (95% CI)	11989 (969–15804)	Raj[37]
			Direct hospital cost	Mean, $ (95% CI)	10639 (8721–13871)	
			Indirect hospital cost	Mean, $ (95% CI)	1119 (696–1728)	
		Coping strategy	Borrowing money from friends or relatives, for surgery	Percentage	49.8	
			Pledging gold, for surgery	Percentage	34	
			Private Loans	Percentage	10.4	
			Borrowing money, after hospital discharge (6 months) for covering expenses and loan repayment	Percentage	52.1	
**Cross-sectional **	India	Financial Hardship	Financial position not at all adequate to look after patient	Percentage	22	Das[39]
			Financial situation very much worsened since patient's illness	Percentage	44	
		OOPP	Total expenditure (Direct +Indirect costs), for ACS treatment	Median, (Min-Max)	7620.95 (818.45–57875.92)	Daivadanam[42]
		Catastrophic Expenditure	Health spending >threshold 40% of household's Capacity to pay	Percentage (95% CI)	84 (79.04, 88.96)	
		Coping strategy	New loans/asset sale vs no loan/asset sale only for ACS treatment	OR (95% CI)	6.97 (1.48,32.85)	
			Exclusively used savings	Percentage	14	
			Solely Loans	Percentage	41	
			Combinations of loans, savings, gifts, insurance	Percentage	37	
		OOPP	Per hospital stay, private + public (1995–1996)	Mean, $	1174.81	Engelgau[25]
			Per hospital stay, private + public 2004	Mean, $	1958.02	
			Per outpatient visit, private+public (1995–1996)	Mean, $	55.47	
			Per outpatient visit, private+public 2004	Mean, $	54.82	
		Catastrophic Expenditure	Patient with CVD vs CDs	OR	1.12(0.99,1.27)	
		Impoverishment	Patient with CVD vs CDs	OR	1.37(1.23,1.53)	
		OOPP	OOPP spending as proportion of total household spending, in high income group	Percentage	39.3	Huffman[44]
			Total expenditure(direct+indirect), among high income group	Mean, $ (95% CI)	2916.8 (1056, 5902)	
		Financial Hardship	Decrease in individual income in high income group	Percentage	25.1	
			Decrease in household income in high income group	Percentage	26.3	
		Catastrophic Expenditure	OOP health spending >40% non-food expenditure in low income group	Percentage	92	
			ACS vs stroke	OR (95% CI)	0.6 (0.37, 0.97)	
			CHE among low income group vs high income group	OR (95% CI)	6.59 (2,23, 19,45)	
		Distress Financing	Distress financing following CVD related hospitalization in low income group	Percentage	64	
			ACS (ref)vs stroke	OR (95% CI)	1.3 (0.21,0.51)	
			Distress financing among low income group vs high income group	OR (95% CI)	1.3 (0.68,2.49)	
**Cross-sectional**		Distress Financing	Borrowings to meet OOPP for inpatient care (Rural/Urban)	Percentage	52/25	Joe[33]
			Contributions/assistance from friends/relatives to meet OOPP for inpatient care (Rural/Urban)	Percentage	27/18	
			Sale assets to meet OOPP for inpatient care (Rural/Urban)	Percentage	8/5	
			CVD vs no CVD for Borrowing to meet OOPP for inpatient care	OR (95% CI)	0.87 (0.87,0.88)	
			CVD vs no CVD for Sale of asset to meet OOPP for inpatient care	OR (95% CI)	1.05 (1.04,1.06)	
			CVD vs no CVD for Contribution/assistance from friend to meet OOPP for inpatient care	OR (95% CI)	1.12 (1.11,1.12)	
		OOPP	Hospital admissions per household member, in affected households (1yr)	Mean, $ (95% CI)	252.61 (259.08, 337.43)	Karan[28]
			Hospital admissions per household member, in match control households (1yr)	Mean, $ (95% CI)	63.21 (52.81, 73.62)	
			Outpatient visits per household member, in affected households (15 days)	Mean, $ (95% CI)	9.22 (8.31, 10.13)	
			Outpatient visits per household member, in match control households (15 days)	Mean, $ (95% CI)	3.99 (3.23, 5.76)	
			OOPP spending as proportion of total household spending, in affected household (15 days)	Percentage (95% CI)	27.22 (25.11, 29.33)	
			OOPP spending as proportion of total household spending, in match control households (15 days)	Percentage (95% CI)	10.72 (9.47, 11.97)	
		Coping strategy	Borrowed or sold assets to pay for inpatient treatment, in affected household	Percentage (95% CI)	32.6 (30.74,34.59)	
			Borrowed or sold assets to pay for inpatient treatment, in matched control household	Percentage (95% CI)	12.8 (11.41,14.20)	
		OOPP	Per hospital stay	Mean, $	869.96	Rao[38]
			Household consumption expenditure, per year	Percentage	30	
		Coping Strategy	Used household savings for hospital treatment	Percentage	57	
			Borrowed for hospital treatment	Percentage	35	
			Selling of assets for hospital treatment	Percentage	8	
	Bangladesh	Impoverishment	Headcount impoverishment impact of OOPP	Percentage	5.88	Hamid[26]
			Average poverty gap	Mean, $	0.018	
			Normalized poverty gap	Percentage	2	
		Distress Financing	Household with Heart Disease vs no Heart Disease	RR (95% CI)	1.22 (1.05–1.42)	Rahman[30]
	Nepal	Catastrophic Expenditure	Health care spending >10% of total household expenditure	Concentration Index (95% CI)	−0.247 (−0.497,0.002)	Saito[31]
			Mean Positive Overshoot (Mean level/Additional payments exceeding >10% threshold of THE)	Percentage	8.3	
			HD vs no HD, among household from poorest quintile	RR (95% CI)	2.24 (1.29, 3.88)	
			HD vs no HD, among household from wealthiest quintile	RR (95% CI)	2.36 (0.83, 6.71)	
		OOPP	Hospitalization expenses per person, in angina treated household (4 weeks)	Mean, $ (95% CI)	0.97 (-0.01, 1.96)	Alam[24]
	Bangladesh		Hospitalization expenses per person, in matched control household (4 weeks)	Mean, $ (95% CI)	0.24 (0.04, 0.45)	
		Catastrophic Expenditure	OOP health spending share of household’s ‘capacity to pay’ at 40% cut-off, in treated household	Percentage (95% CI)	39.4 (35.87, 42.93)	
			OOP health spending share of household’s ‘capacity to pay’ at 40% cut-off, in matched control household	Percentage (95% CI)	35.87 (32.11,39.63)	
		Impoverishment	Impoverishment due to OOP health payments, in treated household	Percentage (95% CI)	12.63 (10.23, 15.03)	
			Impoverishment due to OOP health payments, in matched control household	Percentage (95% CI)	11.82 (9.29, 14.35)	
		Coping strategy	Borrowing or selling assets to finance health expenditure, in treated household	Percentage (95% CI)	46.06 (42.46, 49.66)	
			Borrowing or selling assets to finance health expenditure, in matched control household	Percentage (95% CI)	40.08 (36.23,43.93)	
	India	OOPP	Hospitalization expenses per person, in angina treated household (4 weeks)	Mean, $ (95% CI)	1.46 (0.88, 2.04)	
			Hospitalization expenses per person, in matched control household (4 weeks)	Mean, $ (95% CI)	1.68 (0.35,3.01)	
		Catastrophic Expenditure	OOP health spending share of household’s ‘capacity to pay’ at 40% cut-off, in treated household	Percentage (95% CI)	33 (30.24, 35.76)	
			OOP health spending share of household’s ‘capacity to pay’ at 40% cut-off, in matched control household	Percentage (95% CI)	26.3 (23.38, 29.22)	
		Impoverishment	Impoverishment due to OOP health payments, in treated household	Percentage (95% CI)	10.2 (8.43,11.97)	
			Impoverishment due to OOP health payments, in matched control household	Percentage (95% CI)	8.32 (6.49, 10.15)	
		Coping strategy	Borrowing or selling assets to finance health expenditure, in treated household	Percentage (95% CI)	51.79 (48.86, 54.72)	
			Borrowing or selling assets to finance health expenditure, in matched control household	Percentage (95% CI)	43.56 (40.27, 46.85)	
	Nepal	OOPP	Hospitalization expenses per person, in angina treated household (4 weeks)	Mean, $ (95% CI)	1.18 (0.15, 2.20)	
			Hospitalization expenses per person, in matched control household (4 weeks)	Mean, $ (95% CI)	0.47 (-0.01, 0.94)	
		Catastrophic Expenditure	OOP health spending share of household’s ‘capacity to pay’ at 40% cut-off, in treated household	Percentage (95% CI)	21.27 (17.99, 24.55)	
			OOPP health spending share of household’s ‘capacity to pay’ at 40% cut-off, in matched control household	Percentage (95% CI)	16.75 (13.61, 19.89)	
		Impoverishment	Impoverishment due to OOP health payments, in treated household	Percentage (95% CI)	8.37 (6.15, 10.59)	
			Impoverishment due to OOP health payments, in matched control household	Percentage (95% CI)	6.2 (4.17, 8.23)	
		Coping strategy	Borrowing or selling assets to finance health expenditure, in treated household	Percentage (95% CI)	57.62 (53.66, 61.58)	
			Borrowing or selling assets to finance health expenditure, in matched control household	Percentage (95% CI)	53.6 (49.41, 57.79)	
	SriLanka	OOPP	Hospitalization expenses per person, in angina treated household (4 weeks)	Mean, $ (95% CI)	1.97 (1.48, 2.45)	
			Hospitalization expenses per person, in matched control household (4 weeks)	Mean, $ (95% CI)	0.18 (-0.04, 0.41)	
		Catastrophic Expenditure	OOP health spending share of household’s ‘capacity to pay’ at 40% cut-off, in treated household	Percentage (95% CI)	21.87 (17.34, 26.40)	
			OOPP health spending share of household’s ‘capacity to pay’ at 40% cut-off, in matched control household	Percentage (95% CI)	11.87 (8, 15.74)	
		Impoverishment	Impoverishment due to OOP health payments, in treated household	Percentage (95% CI)	5.31 (2.85, 7.77)	
			Impoverishment due to OOP health payments, in matched control household	Percentage (95% CI)	1.87 (0.25,3.49)	
		Coping strategy	Borrowing or selling assets to finance health expenditure, in treated household	Percentage (95% CI)	21.25 (16.77, 25.73)	
			Borrowing or selling assets to finance health expenditure, in matched control household	Percentage (95% CI)	13.44 (9.36,17.52)	

OR: Odds Ratio, HD: Heart Disease, THE: Total Health Expenditure, CVD: Cardiovascular Disease, OOP: Out of Pocket, CI: Confidence Interval

### Economic impact of diabetes

**Table 3** demonstrates the OOPP for diabetes in India and Pakistan, impoverishing effect in Bangladesh and catastrophic health expenditure led by diabetes treatment in Nepal. Inpatient diabetes care covered 17% of the household expenditure and income respectively in India [38, 45]. The cost of diabetic treatment varied significantly between private and public hospitals (6602.13 USD vs. 1320.43 USD) in India within a year [45]. Khuwaja et al. reported slightly higher direct cost for diabetes care in India as compared to Pakistan [46, 47]. Households with diabetes posed more than twice the risk of spending more than 10% of total expenditure on health than households without diabetes, and the mean positive overshoot was 10.2% [31]. Moreover, 5.25% of households fell into poverty due to payment for diabetes care in Bangladesh, and the poor household falls short of the poverty line by 1.1 cents [26]. Similar to financing for CVDs, in case of diabetes rural households continued to adopt distress financing as compared to the urban household. Selling off assets and assistance from family or friends were respectively 13 times and 21 times more common in diabetes affected households as compared to households without diabetes [33]. **(Table 3)**

**Table 3:** Economic impact of diabetes.

Study Design	Location	Type of Outcome	Outcome Specified as	Assessment Type	Point Estimate	Author
**Cross- sectional**	India	OOPP	Per hospital stay, private + public (1995–1996)	Mean, $	456.87	Engelgau[25]
			Per hospital stay, private + public 2004	Mean, $	783.21	
			Per outpatient visit, private+public (1995–1996)	Mean, $	24.14	
			Per outpatient visit, private+public 2004	Mean, $	41.77	
		OOPP	Direct costs, per year (eg. Drugs, transport, consultations)	Mean, $ (SD)	1103.30 (948.68)	Grover [46]
			Indirect costs, per year (eg. Loss of income, days lost because of illness for patient and caregivers)	Mean, $ (SD)	463.57 (1121.87)	
		Distress Financing	Borrowed to meet OOPP on inpatient care (Rural/Urban)	Percentage	46/26	Joe[27]
			Contributions/assistance from friends/relatives to meet OOP expenditure on inpatient care (Rural/Urban)	Percentage	27/21	
			Sale assets to meet OOPP on inpatient care (Rural/Urban)	Percentage	9/2	
			Diabetes vs no diabetes: Borrowing to meet OOPP for inpatient care	OR (95% CI)	1.01 (1.00,1.01)	
			Diabetes vs no diabetes: Sale of asset to meet OOPP for inpatient care	OR (95% CI)	1.13 (1.11,1.15)	
			Diabetes vs no diabetes: Contribution/assistance from friend to meet OOPP for inpatient care	OR (95% CI)	1.21 (1.20,1.22)	
		OOPP	Mean OOP payment per hospitalization	Mean, $	418.49	Rao[38]
			OPP share of total annual household expenditure	Percentage	17	
		Income	Family income in private hospital, per year	Mean, $	6602.13	Shobhana[45]
			Family income in public hospital, per year	Mean, $	1320.43	
		OOPP	Income spent on DM, by inpatient care	Percentage	17.5	
			Income spent on DM, by outpatient care	Percentage	7.7	
		OOPP	Average cost for each doctor visit	Mean, $ (SD)	10.83 (6.799)	Joshi [28]
	Bangladesh	Impoverishment	Headcount impoverishment impact of OOPP	Percentage	5.25	Hamid[58]
			Average poverty gap	Mean, $	0.011	
			Normalized poverty gap	Percentage	1	
	Pakistan	OOPP	Direct cost, per year	Mean, $	939.88	Khowaja[47]
			Indirect cost, per year	Mean, $	68.18	
	Nepal	Catastrophic Expenditure	Household spending >10% of total expenditure on health care	Concentration Index (95% CI)	0.099(-0.304,0.107)	Saito[31]
			Mean Positive Overshoot (Mean level/Additional payments exceeding >10% threshold of THE)	Percentage	10.2	
			Diabetes vs no diabetes, among household from poorest quintile	RR (95% CI)	2.37 (1.16, 4.83)	
			Diabetes vs no diabetes, among household from wealthiest quintile	RR (95% CI)	0.45, 2.39)	

DM: Diabetes Mellitus, SD: Standard Deviation, THE: Total Household Expenditure

### Economic impact of cancer

**Table 4** shows the studies on the economic impact of cancer from India, Bangladesh, and Pakistan. The mean inpatient expenditure in cancer affected household was almost 5 times higher than the matched control household in India (326.93 USD vs 66.42 USD) [29]. Whereas another study in India reported very high cost of treatment alone of around USD 2543 [48] and the cost of hospitalization was reported to be more than double in 8 years span [25]. Seven out of ten households in Pakistan perceived breast cancer the imposed financial burden, and the cost of treatment was unmanageable for breast cancer [41]. Studies reported significant impoverishment induced by cancer treatment among households in Bangladesh and India [25, 26]. In Bangladesh, the impoverishing effect was much more pronounced for the most impoverished family as they further fall into poverty by 8% due to cancer treatment. This is the highest reported normalized poverty gap as compared to CVD and Diabetes by the same study. Similar to other NCDs, households borrowed or sold the asset to finance for inpatient care as compared to matched or control households [33]. **(Table 4)**

**Table 4:** Economic impact of cancer among household in South Asia.

Study Design	Location	Type of Outcome	Outcome Specified as	Assessment Type	Point Estimate	Author
**Cross- sectional**	India	OOPP	Per hospital stay, private + public (1995–1996)	Mean, $	1044.28	Engelgau [25]
			Per hospital stay, private + public 2004	Mean, $	2349.62	
			Per outpatient visit, private + public (1995–1996)	Mean, $	78.32	
			Per outpatient visit, private + public (1995–1996)	Mean, $	110.95	
		Catastrophic Expenditure	Patients with Cancer versus CDs	OR (95% CI)	2.7 (2.10, 3.10)	
		Impoverishment	Patients with Cancer versus CDs	OR (95% CI)	2.33 (1.86, 2.91)	
		Distress Financing	Borrowed for financing inpatient care (Rural/Urban)	Percentage	60/37	Joe[27]
			Contributions/assistance from friends/relatives for financing inpatient care(Rural/Urban)	Percentage	32/19	
			Sale assets for financing inpatient care(Rural/Urban)	Percentage	14/10	
			Cancer vs no cancer: Borrowing for inpatient care	OR (95% CI)	1.11 (1.10,1.12)	
			Cancer vs no cancer: Sale of asset for financing inpatient care	OR (95% CI)	1.33 (1.32,1.34)	
			Cancer vs no cancer: Contribution from friends/relatives for financing inpatient care	OR (95% CI)	1.29 (1.28,1.3)	
		OOPP	Inpatient OOPE, per member in cancer affected household (1year)	Mean, $	326.93 (277.87, 375.99)	Mahal[29]
			Inpatient OOPE, per member in matched control household (1year)	Mean, $	66.42 (43.21, 89.69)	
			Non-medical consumption expenditure, per member in cancer affected household (15days)	Mean, $ (95% CI)	18.09(18.53, 21.05)	
			Non-medical consumption expenditure, per member in cancer affected household (15days)	Mean, $ (95% CI)	19.76 (18.53, 21.05)	
		Coping Strategy	Borrowing or selling assets to finance inpatient care in cancer affected household	Percentage (95% CI)	51.4(47.98, 54.82)	
			Borrowing or selling assets to finance inpatient care in matched control household	Percentage (95% CI)	15.77(13.28, 18.26)	
		OOPP	Cost of investigation	Mean, $	1030.42	Nair[48]
			Cost of treatment	Mean, $	2543.02	
			Indirect cost	Mean, $	1677.33	
			Opportunity cost	Mean, $	1118.20	
		Hardship	Faced financial hardship	Percentage	75	
		Coping Strategy	Family saving	Percentage	36.5	
			Borrowings	Percentage	39.12	
			Sales of assets (land, cattle, ornament, etc.)	Percentage	12.27	
			Medical reimbursement/ health insurance	Percentage	6.22	
			Other assistance (Government/philanthropic)	Percentage	5.89	
	Bangladesh	Impoverishment	Headcount impoverishment impact of OOPP	Percentage	25	Hamid[58]
			Average poverty gap	Mean, $	0.068	
			Normalized poverty gap	Percentage	8	
	Pakistan	Hardship	Cost more than anticipated	Percentage	70	Zaidi[41]
			Perceived level of burden unmanageable	Percentage	70	

CDs: Communicable diseases

### Economic impact of chronic respiratory diseases

**Table 5** summarizes the studies conducted in India, Bangladesh, and Nepal on OOPP, catastrophic health expenditure, impoverishment and financial coping strategy adopted for treatment of chronic respiratory diseases. The average out of pocket expenditures per visit for non-domiciliary treatment of COPD was higher for urban households in Bangladesh than the rural ones (41.98 USD vs 4.38 USD) [49]. Almost 6% of the Bangladesh household fell into poverty due to payment for asthma health care services, and the intensity of medical impoverishment was increased by 2% for the poorest household. The risk of household undergoing catastrophic health expenditure was higher in the poorest household as compared to wealthiest quintile, RR 2.09(1.39 at 95% CI) in Nepal. Around three-quarters of Bangladeshi household were at risk of implementing one of the coping strategies like borrowing and selling off assets to finance for Asthma treatment [30], and this risk was higher among urban households [49]. Among Indian households the OOPP per inpatient treatment in private and public hospital for bronchial asthma was increased tremendously between 1995 and 2004; 195.80 USD and 522.13 USD respectively [25]. **(Table 5)**

**Table 5:** Economic impact of chronic respiratory diseases among household in South Asia.

Study Design	Location	Type of Outcome	Outcome Specified as	Assessment Type	Point Estimate	Author
**Cross-sectional**	India	OOPP	Per hospital stay, private + public (1995–1996)	Mean, $	195.80	Engelgau[25]
		OOPP	Per hospital stay, private + public 2004	Mean, $	522.13	
			Per outpatient visit, private + public (1995–1996)	Mean, $	20.88	
		** **	Per outpatient visit, private + public 2004	Mean, $	33.93	
	Bangladesh	Impoverishment	Headcount impoverishment impact of OOPP	Percentage	5.89	Hamid[58]
			Average poverty gap	Mean, $	0.018	
			Normalized poverty gap	Percentage	2	
		Distress Financing	Household with Asthma vs no Asthma	RR (95% CI)	1.73 (1.35–2.22)	Rahman[30]
		Financial Hardship	Prevalence of economic consequences (Rural/Urban)	Percentage	2.4/12.5	Uddin[60]
		OOPP	OOPE per visit for seeking outpatient treatment for COPD Urban	Mean, $	41.98	
			OOPE per visit for seeking outpatient treatment for COPD Rural	Mean, $	4.38	
		Coping Strategy	Sold household assets (Rural/Urban)	Percentage	0.3/1.1	
			Spent/reduced savings(Rural/Urban)	Percentage	0.0/4.3	
			Reduced expenditure on food (Rural/Urban)	Percentage	0.7/6.5	
			Borrowed money from relative/friend (Rural/Urban)	Percentage	1/7.1	
	Nepal	Catastrophic Expenditure	Household spending >10% of total expenditure on health care	Concentration Index (95% CI)	−0.185 (−0.389 to 0.018)	Saito[31]
			Mean Positive Overshoot (Mean level/Additional payments exceeding >10% threshold of THE)	Percentage	12.3	
			Asthma vs no Asthma, among household from poorest quintile	RR (95% CI)	2.09 (1.28, 3.42)	
			Asthma vs no Asthma, among household from wealthiest quintile	RR (95% CI)	1.39 (0.40, 4.82)	

THE: Total Household Expenditure

## Discussion

This systematic review summarizes 22 studies assessing the economic impact in terms of OOPP, catastrophic health expenditure, impoverishment caused by management and treatment of CVDs, diabetes, cancer, chronic respiratory diseases among households in South Asia and their financial coping strategy. Households suffering from NCDs had higher out of pocket expenditure, catastrophic health expenditures and were more likely to undergo impoverishment compared to its counterparts without NCDs. The review also pointed out that the current health services for these NCDs are unaffordable to already poverty-stricken population of the region. The most common coping strategy adopted by South Asian household were borrowing and selling off assets. Finally, it could be shown, that there is a lack of studies on the economic impact of the specific type of cancer and COPD in the South Asian region.

Before discussing the major findings of this systematic review, it is important to discuss on the methodological variations the studies have presented in measuring OOPP, catastrophic health expenditure, impoverishment across South Asian countries. The methodological differences occurred in the measurement of out of pocket payment i.e. inclusion of direct cost, indirect cost, non-medical cost and variance in recall period. Though a majority of the studies were based on random samples, the cost associated with NCDs was self-reported in all cases. These self-reported costs associated with NCDs even for random samples are likely to over-report the expenses specially in lack of comparative group [16, 50]. The recall period in the studies varied from a few days to 12 months. The longer recall period is subjected to misreporting due to respondent inability to remember exact out of pocket expenditures while short recall period does not capture the actual expenses and are likely to exaggerate or over report the expenses [51, 52].

Currently, WHO uses the incidence of catastrophic health expenditures and the incidence of impoverishment due to out-of-pocket health payments as indicators to monitor the level of financial protection for Universal Health Coverage [53]. However, in this review majority of the studies reported OOPP due to NCDs and only few studies reported the incidence of catastrophic health expenditure and even fewer (only three) studies reported incidence of impoverishment. Hence restressing the gap of availability of data regarding financial protection in low and middle-income countries [53] including the South Asian region. Likewise, for measurement of catastrophic health expenditure studies used different thresholds ranging from 10% and 40% spent as health expenditure of total household consumption expenditure or total household non-food consumption expenditure while one study used “mean catastrophic positive overshoot” i.e. the degree by which the average out of pocket expenditure by households that have experienced catastrophe has exceeded the given catastrophic threshold [51].Thus, hindering the comparability between studies and diseases.

Similarly, approaches to assess impoverishment among the studies differed widely. Studies either used absolute poverty line or locally derived poverty line while only one study assessed poverty gap (i.e. households pushed further into poverty). Moreover, the OOPP, catastrophic health expenditure and impoverishment are out-product of political and societal settings: availability and access to health services, risk pooling and health financing mechanism and poverty levels in each country. Hence results should be cautiously interpreted on these socio-political paradigms [51, 54]. Thus, this systematic review highlights the need for standardized definitions, thresholds for assessing OOPP and its impact, studies going beyond the measurement of OOPP alone and measuring the incidence of catastrophic health expenditure and impoverishment. Along with this, preparing tools that are not sensitive to political and societal factors is must to make a cross-country and fair comparisons.

Additionally, most of the studies were cross-sectional, hence, failed to answer whether the catastrophic and impoverishing effects observed, and coping strategy adopted occurred in a unit of time or is the aggregation of such impacts over a period for a household. The duration over which a household undergoes catastrophic or impoverishing effects may be more important than the incidence of the results in the population itself [55] specially in case of NCDs which require lifelong expenses for medication and care which was not reported in any of the studies in the review. Moreover, majority of the studies in the review were of poor quality mainly due to inadequately defined NCDs, lack of reference group/comparator and cross section nature of the study **(S2 Table).** Out of pocket expense for NCDs in lack of reference group or comparator gives very little information. Hence, these findings stress the need of robust research on NCDs and its economic impact with optimal methodological design along with appropriate reference group and comparators group to facilitate the production of meaningful and comparable national and regional estimates.

The trends of economic impact of NCDs and reasons can be presented and discussed, even though the methodological differences are present. Firstly, the review reconfirmed that households suffering from NCDs had higher out of pocket expenditure, catastrophic health expenditures and were more likely to undergo impoverishment compared to its counterparts which concurs with similar literature review conducted in low and middle-income countries [56, 57]. A literature review on the financial burden on NCDs in resource constraint setting showed that comorbidities associated with NCDs and the cost of medication occupied the largest proportion in direct cost associated with treatment of NCDs [16, 58].

Secondly, the review also pointed out that the current health services for these NCDs are unaffordable to already poverty-stricken population of the region. For instance, among the studies in the review, the highest out of pocket direct cost 11,989 USD was reported for congenital heart surgery for 0 to 18 years children [37] in India where almost a quarter of the population live below 2 USD per day [5]. Similar disproportionate risk of catastrophic expenditure among uninsured and poor household was also seen in case of chronic respiratory disease [26, 31] and CVDs [25, 35] in the review. Surprisingly, the consequential effect was also visible in the high-income household [31, 44]. For instance, the households with heart disease from wealthiest quintile in Nepal had slightly increased risk of catastrophic health expenditure than the poorest household. This does not necessarily mean that the poorest household suffer less from CVDs than richest household. This may also signify that poor household does not have financial ability to seek care, so they avoid health service hence lesser expenditure altogether. However, households or individuals not seeking health care for NCDs and its financial implications was overlooked and not discussed in any of the studies included in the review.

Thirdly, this systematic review also showed that borrowing and selling off assets as the most common coping strategy adopted by households in the region for all major NCDs in this review. A study done among African nations showed similar results for paying their inpatient health costs [59]. However, the proportion of households adopting coping strategy varied inconsistently between the rural or urban place of residence [27, 60]. On the one hand, this difference in coping behavior can be attributed to poor economic conditions where only well-endowed households can pay for their health care services and people from the rural area must find alternative measures to pay for their health [61]. Whereas the high percentage of urban distress financing reiterates that coping behavior is strongly correlated with the availability of social capital, valuable assets, possibility of getting a loan which is higher among affluent group living in a urban household [59, 62]. High dependence on coping strategy at present will reduce the ability of families to deal with unprecedented health shocks in the future and increase debt in a poor household [57]. Furthermore, borrowing or incurring loan or contributions from family and friends may also depend on the individual needing health care. The healthcare need of female and elderly are not prioritized in the patriarchal society as such of South Asia, hence reduced coping measures or not seeking health care at all [25]. This intersectionality of gender and age group from the perspectives of health financing and coping strategies among households has not been studied yet in low and middle-income countries [27].

Fourthly, one of the peculiar findings of the review is the lack of studies on the economic impact of specific type of cancer and COPD in South Asian region. One the explanations for this could be insufficient population-based cancer registry in the region to draw cancer-specific data [63]. It is commonsensical that disregarding different types, stages and trajectories of cancer will lead to underreporting and underestimation of the financial burden caused by cancer [64, 65]. We identified 14 studies from India, 1 study from Nepal, 2 studies from Pakistan, 2 studies from Bangladesh and 1 study from India, Bangladesh, Sri Lanka and Nepal combined, which allows in-depth information from South Asian countries. The studies from Bangladesh, Nepal and Sri Lanka were absent in previously conducted systematic review on global impact and impoverishment of NCDs [18]. However, studies from Maldives and Bhutan were still missing. This lack of evidence may be subjected to the fact that NCDs are emerging public health problem in South Asia region where the health system is predominantly focused on tackling the challenges caused by infectious disease [7, 66]. Similar lack of studies assessing the economic impact of COPD could be because COPD is ignored as cough or smoker cough. This leads to reduced number of individual seeking health service but potentially increase costs due to ill-diagnosis or later diagnosis. As the epidemiological burden of COPD increases in the region with aging population [67], it can be expected that household will undergo higher out of pocket payment and its subsequent impact. Hence, future studies on the economic impact of COPD in South Asia could provide us with crucial information.

Thus, given the lack of risk pooling mechanism, heavy dependence on paying out of pocket for health financing followed by rapid privatization of health services in the region [8] and preference of private health facilities over public facilities for quality of care and diagnostics in case of NCDs [68]; it is very likely that seeking health care services for NCDs will push households to medical poverty and will create the intergenerational cycle of poverty and poor health [8, 54]. If the current situation prevails it will also undermine the goal of attainment of Universal Health Coverage- appropriate care at affordable cost in the South Asian region. There has been some initiatives from South Asian countries to bring forward population-based insurance scheme [69–71], WHO Package of Essential Non-communicable program [72, 73]; however, challenges remain. Recent evidences suggest that population covered under health insurance program or national schemes is not an ultimate solution for financial protection [74, 75]. In order to extend the financial protection national programs should be based on mechanisms where the large share of health expenditure is prepaid through taxation or mandatory payment system [74–76]. Likewise, pro-poor programs to eliminate financial barriers in uptake and adherence to cost effective interventions needs to be prioritized [76]. This is however profoundly absent in the South Asian region. Thus, in such a scenario the appropriate mix of preventive and promotive approaches to modify NCDs risk factors and reduce the epidemiological burden hence reducing the cost associated with its treatment and management in the long run could be beneficial.

One of the biggest strength of this systematic review was the comprehensive nature of search strategy applied and use of Newcastle–Ottawa Quality Assessment Scale to assess the quality of non-randomized studies including case-control and cohort studies [77]. This has been previously tested in a systematic review done to evaluate the global impact of NCDs on households and impoverishment with appropriate adaptation as per the objectives of this systematic review [18]. Another significant strength of the review is the conversion of local currency to US Dollars. The Purchasing Power Parity conversion rate is the number of units of a country's currency required to buy the same amounts of goods and services in the domestic market as U.S. dollar would buy in the United States [78]. This conversion factor takes account of the GDP of the country hence giving superior comparability than exchange rate. Moreover, all USD converted to dollars in 2016 through consumer price index conversion takes inflation rate in consideration hence inferences on out of pocket payment are comparable and reliable. Most importantly, this systematic review provides the much-needed evidence assessing the economic impact of NCDs, identifying the gaps in evidence gaps and understanding areas for further research exclusively for South Asia.

The main limitation of our review is the use of only two databases, Medline and Embase for searching articles though they cover a broad range of peer reviewed articles published from 1946 to till date on biomedicine and health. Hence, this review may have missed related articles from other databases. In order to minimize this limitation, we also conducted snowballing of references to ensure no potentially relevant studies were left out. But we acknowledge that relevant publications in local languages and ministerial surveys and reports regarding this issue which could have been of vital importance has not been included in the review. Another major limitation of this review occurred in the selection of cancer studies. We only included four cancers with leading DALYs rates among men and women in South Asian region in the search strategy for detail exploration. However, we widened out our inclusion criteria so that studies assessing economic impact but failing to mention non-specific cancers/neoplasms was also included in the review so that we do not miss out important information on cancer led economic impact. Lastly, our review does not take in account of comorbidities associated with NCDs which have found to play a significant role in increasing disease burden and cost of treatment.

## Conclusion

Our review suggests that the economic impact of CVDs, diabetes, cancer and chronic respiratory diseases among households in South Asia seems dire. Out of pocket payment, catastrophic payment and impoverishment are significantly high in households with NCDs and affects households in all income levels. Borrowing and selling off assets were most common coping behavior exhibited by South Asian household and differed inconsistently with rural and urban residence. However, the studies on economic impact associated with NCDs specially assessing catastrophic health expenditure and impoverishment are inadequate in the region and the gap of evidence for COPD and specific cancer is even higher. Thus, the review highlights the need for robust research on economic impact of NCDs so that evidence-informed nationally tailored prepayment mechanisms covering NCDs can be developed. The review also calls for standardization of tools measuring out of pocket payment and associated catastrophic and impoverishing effect in South Asia which will facilitate the production of meaningful and comparable national and regional estimates.

## Supporting information

S1 Table Detail search strategy. (DOCX)

S2 Table Risk of bias analysis. (DOCX)

## Abbreviations

COPD: Chronic Obstructive Pulmonary Diseases
CVDs: Cardiovascular Diseases
DALYs: Daily Adjusted Life Years
GDP: Gross Development Product
NCDs: Non-Communicable Diseases
NOS: Newcastle–Ottawa Quality Assessment Scale
OOPP: Out of Pocket Payment
SDGs: Sustainable Development Goals
USD: United States Dollar
WHO: World Health Organization
