# Correction: The economic impact of non-communicable diseases among households in South Asia and their coping strategy: A systematic review

**DOI:** 10.1371/journal.pone.0211588

**Published:** 2019-01-25

**Authors:** Anupa Rijal, Tara Ballav Adhikari, Jahangir A. M. Khan, Gabriele Berg-Beckhoff

An incorrect number appears throughout the article. This systematic review includes 22 articles in total, but in three sentences the number 21 appears incorrectly. This error first appears in the first sentence of the Methods Section of the Abstract. This error also appears in the first sentence under the subheading “Characteristics of the study reviewed” in the Results section. Lastly, this error appears in the fifth sentence of the fifth paragraph under the same subheading in the Results section.

There are incorrect numbers in Fig 1. In the box labelled “Full Text articles excluded,” the total number of articles should be 24, not 25. In the same box, the total number of articles under “No or non-specified NCD” should be 15, not 14. Please see the corrected Fig 1 here.

**Fig 1:**
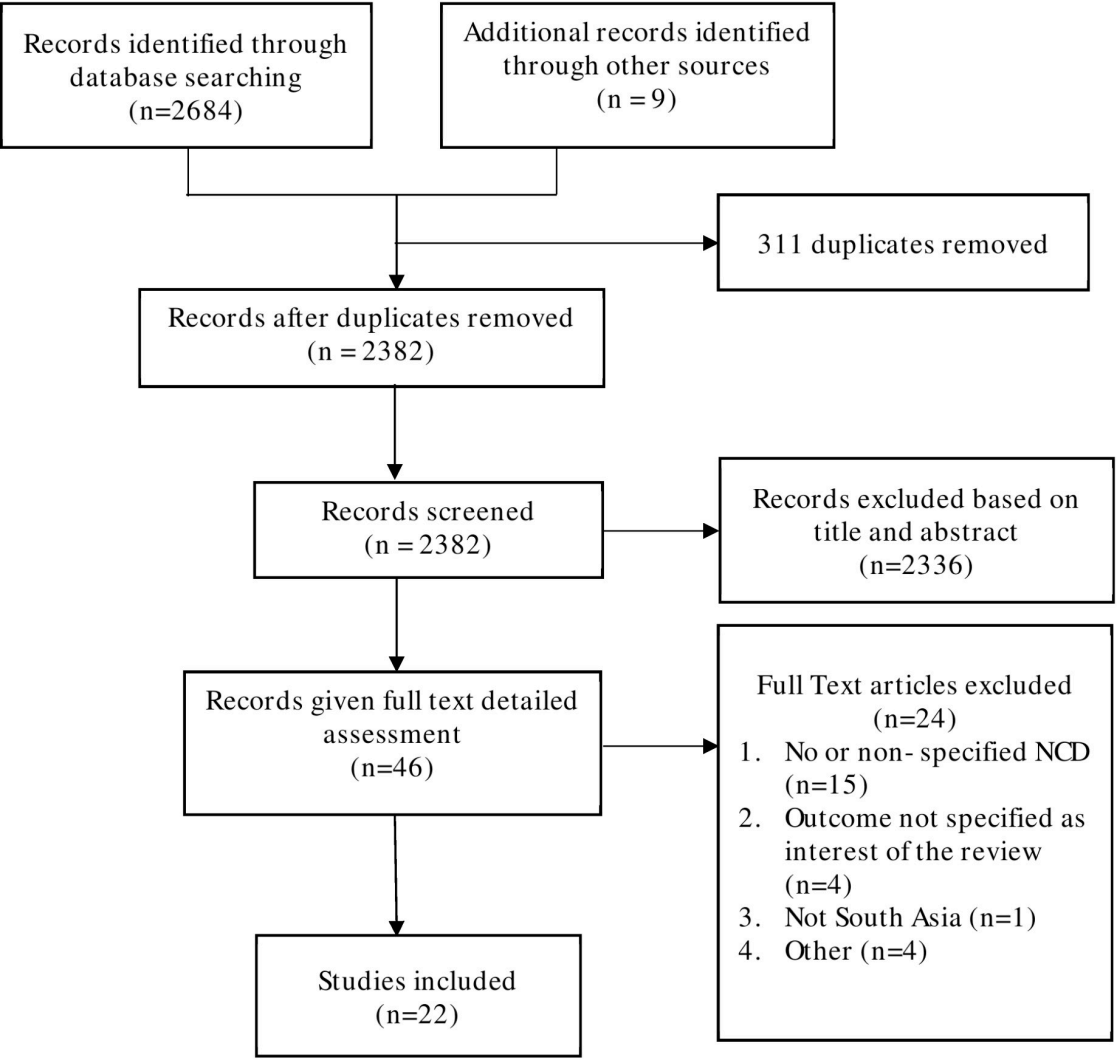
PRISMA flow-chart for systematic review of studies.
