# Supplementary material for: The economic impact of non-communicable diseases among households in South Asia and their coping strategy: A systematic review
Source: PLoS One. 2018 Nov 21;13(11):e0205745. doi: 10.1371/journal.pone.0205745 (PMC6248902; doi:10.1371/journal.pone.0205745)
Supplement: S1 Table — (DOCX) [file pone.0205745.s001.docx]

**Table 1: Detail search strategy**

| **S.N.** |  | **Embase 1974 to 2017 February 06*** | **No. of articles as of February 17** | **Medline 1946 to February Week 2 2017*** | **No. of articles as of February 17** |
| --- | --- | --- | --- | --- | --- |
|  |  | **Exposure(E)** |  |  |  |
| 1 | Cardiovascular disease | exp non communicable disease/ or exp cardiovascular disease/ or exp non insulin dependent diabetes mellitus/ or exp respiratory tract disease/ or exp lung cancer/ or exp breast cancer/ or exp mouth cancer/ or exp uterine cervix cancer/ | 5768972 | exp Diabetes Mellitus, Type 2/ or exp Cardiovascular Diseases/ or exp Respiratory Tract Diseases/ or exp Lung Neoplasms/ or exp Breast Neoplasms/ or exp Mouth Neoplasms/ or Uterine Cervical Neoplasms/ | 3598938 |
| 2 |  | (‘non communicable’ or noncommunicable or ‘non-communicable’).ab,ti | 6775 | non communicable disease.mp. or ('non communicable' or noncommunicable or 'non-communicable').ab,ti | 4225 |
| 3 |  | ((heart or cardiac or cardial or cardiopath* or cardiomyopath* or coronor* or myocord*) adj3 (ischem* or ischaem* or anoxia or hypoxia)).ab,ti | 74684 | ((heart or cardiac or cardial or cardiopath* or cardiomyopath* or coronor* or myocord*) adj3 (ischem* or ischaem* or anoxia or hypoxia)).ab,ti | 48318 |
| 4 |  | (coronary adj3 (insufficien* or occlus* or disease* or acute or atherosclero* or orteriosclero* or sclero* or cordiosclero* or constrict* or vasoconstrict* or obstruct* or stenosis* or thrombo*)).ab,ti | 257369 | (coronary adj3 (insufficien* or occlus* or disease* or acute or atherosclero* or orteriosclero* or sclero* or cordiosclero* or constrict* or vasoconstrict* or obstruct* or stenosis* or thrombo*)).ab,ti | 165866 |
| 5 |  | ((heart or myocard* or cardiac or cardial) adj3 infarct*).ab,ti | 240873 | ((heart or myocard* or cardiac or cardial) adj3 infarct*).ab,ti | 159606 |
| 6 |  | ((cerebrovascul* or brain or 'cerebral vascular' or cerebrovascular) adj3 (accident* or lesion or attack or ischem* or ischaem* or insult* or insuffucien* or arrest* or apoplex*)).ab,ti |  | ((cerebrovascul* or brain or 'cerebral vascular' or cerebrovascular) adj3 (accident* or lesion or attack or ischem* or ischaem* or insult* or insuffucien* or arrest* or apoplex*)).ab,ti | 29916 |
| 7 |  | (cva or stroke or angina*) .ab,ti | 349266 | (cva or stroke or angina*) .ab,ti | 207808 |
| 8 | Chronic respiratory disease | (chronic and (obstruct* adj3 (lung* or pulmonor* or airway* or bronch* or respirat*))).ab,ti | 16877 | (chronic and (obstruct* adj3 (lung* or pulmonor* or airway* or bronch* or respirat*))).ab,ti | 10947 |
| 9 | Cancer (Lung(M), oral(M), Breast(F), Cervical(F),) 4 most common cancer in South Asia among men and women | ((lung* or pulmonar* or oral* or mouth* or breast* or mamma*) adj3 (neoplas* or cancer* or carcino* or adenocarcino* or metasta* or sarcom*)).ab,ti | 633910 | ((lung* or pulmonar* or oral* or mouth* or breast* or mamma*) adj3 (neoplas* or cancer* or carcino* or adenocarcino* or metasta* or sarcom*)).ab,ti | 412264 |
| 10 |  | ((cervix or cervical) adj3 (cancer* or neoplas* or tumo* or carcinom* or malign*)).ab,ti | 81809 | ((cervix or cervical) adj3 (cancer* or neoplas* or tumo* or carcinom* or malign*)).ab,ti | 59377 |
| 11 | Diabetes | (('adult onset' or 'type 2' or 'type ii' or 'non-insulin dependent' or 'noninsulin dependent' or 'insulin independent') adj3 diabet*).ab,ti |  | (('adult onset' or 'type 2' or 'type ii' or 'non-insulin dependent' or 'noninsulin dependent' or 'insulin independent') adj3 diabet*).ab,ti | 101571 |
| 12 |  | 1 or 2 or 3 or 4 or 5 or 6 or 7 or 8 or 9 or 10 or 11 | 6008833 | 1 or 2 or 3 or 4 or 5 or 6 or 7 or 8 or 9 or 10 or 11 | 3780716 |
|  |  | **Outcome(O)** |  | **Outcome(O)** |  |
| 13 |  | *"cost of living"/ or *budget/ or exp financial deficit/ or *income/ or *"health care cost"/ or *"hospital cost"/ or exp insurance/ or *"cost of illness"/ or exp socioeconomics/ | 546872 | exp Economics/ or exp Health Expenditures/ or *Income/ or *Budgets/ or *Hospital Costs/ or exp Socioeconomic Factors/ or *"cost of illness"/ or *Hospital Costs/ or exp Insurance/ or *Health Care Costs/ | 1244744 |
| 14 |  | ((cost* or econom* or expen*) adj6 (living or individu* or famil* or personal* or patient* or illness* or direct* or indirect*)).ab,ti | 135418 | ((cost* or econom* or expen*) adj6 (living or individu* or famil* or personal* or patient* or illness* or direct* or indirect*)).ab,ti | 80147 |
| 15 |  | (budget* or deficit* or debt* or income or insurance* or 'health insurance' or socioeconom* or pover* or impover* or poor or wealth or 'co-insurance' or coinsurance or copayment or 'user charge' or 'user payment' or 'user fee' or 'direct cost' or 'indirect cost' or 'loan' or 'cost sharing' or 'informal payment' or 'health care payment' or 'health care spending' or deductible or 'spending for health' 'paying for hospital care' or 'paying for health care').ab,ti | 1129127 | (budget* or deficit* or debt* or income or insurance* or 'health insurance' or socioeconom* or pover* or impover* or poor or wealth or 'co-insurance' or coinsurance or copayment or 'user charge' or 'user payment' or 'user fee' or 'direct cost' or 'indirect cost' or 'loan' or 'cost sharing' or 'informal payment' or 'health care payment' or 'health care spending' or deductible or 'spending for health' 'paying for hospital care' or 'paying for health care').ab,ti | 741780 |
| 16 |  | exp family/ or *home/ or *household/ or (famil* or home or household* or personal).ab,ti. | 1819467 | exp Family/ or household.mp or (famil* or home or household* or personal).ab,ti. | 1246706 |
| 17 |  | ('caregiver burden' or microeconom* or 'out of pocket payment' or OOPP or 'out of pocket payments' or 'out-of -pocket-payment' or 'out of pocket' or 'willingness to pay' or 'coping mechanism' or 'coping strategy' or 'selling of assets' or poor* or meagerness or 'Poverty line').ab,ti | 940693 | ('caregiver burden' or microeconom* or 'economic burden' or 'financial burden' or 'out of pocket payment' or OOPP or 'out of pocket payments' or 'out-of -pocket-payment' or 'out of pocket' or 'willingness to pay' or 'coping mechanism' or 'coping strategy' or 'selling of assets' or poor* or meagerness or 'Poverty line').ab,ti | 605971 |
| 18 |  | (catastroph* adj3 (spend* or expend*)).ab,ti | 351 | (catastroph* adj3 (spend* or expend*)).ab,ti | 243 |
| 19 |  | (Value* adj2 'statistical life').ab,ti | 67 | (Value* adj2 'statistical life').ab,ti | 47 |
| 20 |  | 13 or 14 or 15 | 1583861 | 12 or 13 or 14 | 1480137 |
| 21 |  | 16 or 17 or 18 or 19 | 2658367 | 16 or 17 or 18 or 19 | 1796443 |
| 22 |  | 20 and 21 | 795524 | 20 and 21 | 582874 |
|  |  | **Population(P)** |  | **Population** |  |
| 23 |  | exp south asia/ or (Asia or Nepal or India or Pakistan or Bangladesh or 'Sri Lanka' or Bhutan or Maldives or 'South Asian Association for Regional Cooperation' or SAARC or 'SAARC countries').ab,ti | 261519 | (Asia or Nepal or India or Pakistan or Bangladesh or 'Sri Lanka' or Bhutan or Maldives or 'South Asian Association for Regional Cooperation' or SAARC or 'SAARC countries').ab,ti | 115524 |
|  |  | **Study Design** |  |  |  |
| 24 |  | exp "radomized controlled trial (topic)"/ or *cohort analysis/ or *case control study/ or *cross-sectional study/ or exp ecology/ or exp ecosystem monitoring/ or exp model/ or ((random* adj3 (trial* or control)) or rct* or cohort* or 'case control' or 'cross sectional' or ecolog* or ecosystem* or model*).ab,ti | 5059808 |  |  |
|  |  | Exposure and Outcome and Population (Embase and Medline together) **12 and 22 and 23 and 24** | 1557 | Exposure and Outcome and Population (Embase and Medline together) **12 and 22 and 23** | 1369 |
|  |  | (abstracts and english language and publication yr="2000 - 2016") | 1495 | (abstracts and english language and publication yr="2000 - 2016") | 1189 |

*This is detailed search strategy run as of February 17, 2017, based on PECO framework for Embase and Medline (accessed through Ovid) with the number of corresponding articles retrieved for each categories.
