# Supplementary material for: The economic impact of non-communicable diseases among households in South Asia and their coping strategy: A systematic review
Source: PLoS One. 2018 Nov 21;13(11):e0205745. doi: 10.1371/journal.pone.0205745 (PMC6248902; doi:10.1371/journal.pone.0205745)
Supplement: S2 Table — (DOCX) [file pone.0205745.s002.docx]

**Table 1: Risk of bias analysis**

| **Study Design** | **Location** | **Selection** | | | | **Comparability** | **Exposure** | | | **Quality score** | **Author** |
| --- | --- | --- | --- | --- | --- | --- | --- | --- | --- | --- | --- |
|  |  | NCD Definition | Case representativeness | Reference group comparison | Reference definition | Comparability with design or analysis | Ascertainment of exposure | Method of ascertainment | Non-response rate |  |  |
| **Cohort** | India | a* | c | c | c | a* | a* | c | d | 3 | Jan |
|  |  | a* | b* | c | c | b* | a* | c | d | 4 | Kwatra |
|  |  | a* | b* | c | c | b* | a* | c | b | 4 | Raj |
| **Cross-sectional** | India | b | b* | a* | a* | b* | a* | a* | a* | 7 | Engelgau |
|  |  | a* | a* | a* | a* | b* | a* | a* | a* | 8 | Karan |
|  |  | a* | a* | a* | a* | b* | a* | c | d | 6 | Davidnam |
|  |  | a* | a* | c | c | b* | a* | c | a* | 5 | Huffman |
|  |  | b | c | c | c | b* | d | c | d | 1 | Rao |
|  |  | a* | a* | c | c | b* | a* | c | d | 4 | Grover |
|  |  | b | a* | c | c | b* | a* | c | b | 3 | Shobhana |
|  |  | a* | a* | a* | a* | b* | a* | a* | d | 7 | Joe |
|  |  | a* | a* | a* | a* | b* | a* | a* | a* | 8 | Mahal |
|  |  | b | a* | c | c | NA | a* | c | a* | 3 | Das |
|  |  | b | c | c | c | NA | d | c | a* | 1 | Joshi |
|  |  | a* | c | c | c | NA | a* | c | a* | 2 | Nair |
|  | Bangladesh | a* | c | a* | a* | b* | a* | a* | d | 6 | Uddin |
|  |  | b | a* | a* | a* | b* | a* | c | a* | 6 | Rahman |
|  |  | b | a* | a* | a* | b* | a* | a* | d | 6 | Hamid |
|  | Pakistan | a* | c | c | c | b* | a* | c | a* | 4 | Zaidi |
|  |  | a* | b* | c | c | b* | a* | c | b | 4 | Khowaja |
|  | Nepal | a* | a* | a* | a* | b* | a* | c | a* | 7 | Saito |
|  | Nepal, Srilanka, Bangladesh, India | a* | a* | a* | a* | b* | a* | a* | a* | 8 | Alam |
|  | Total | 15 | 16 | 10 | 10 | 19 | 20 | 7 | 11 |  |  |
